# The Medical Profession of Ancient Times: An Anniversary Discourse, Delivered before the New York Academy of Medicine, Nov. 7, 1855

**Published:** 1856-09

**Authors:** 


### BOOK NOTICES.

*The Medical Profession of Ancient Times: an Anniversary Discourse, delivered before the New York Academy of Medicine, Nov.* 7, 1855. By JOHN WATSON, M.D., Surgeon to the New York Hospital. Published by order of the Academy, by Baker & Godwin, New York, 1856.

The work indicated by the foregoing title, although presented to the Academy of Medicine as an Anniversary Discourse, is, nevertheless, a handsomely executed octavo volume of 222 pages.

It is the result of much patient and careful research on the part of the author, and constitutes one of the most valuable histories of Ancient Medicine which has yet been published. In his introductory remarks the author says truly that:—

"To the initiated, Medicine is something more than a profession—it is a world within itself. It has its history, its philosophy, its politics, its literature, of which the world at large knows nothing. It has its subsidiary arts and occupations. It has its organizations and institutions, its ranks and grades of honor. It has its polemics and dissensions, not always amenable to logic or to the learning of the schools. In ethics, traditions, and superstitions, it is older than the church. In use before the civil law, it recognizes no arbitrary enactments: nature is its only court of equity. And who of us shall forget its ever-living charities; its moving scenes of joy and sadness; its many sunny aspects; its benignant, ennobling, liberalizing influences; which few beyond our own circle can properly appreciate, and none so well understand as ourselves!

"No wonder, then, that the members of our profession, drawn together by these hallowed ties, should be disposed to band together as a brotherhood. Such has always been their course. The Druids of early Gaul and Briton, the Asclepiadæ of Greece, the priests of ancient Egypt, the lamas of Central Asia, the Vaidhyas of India, the fraternities of the middle ages, and up to the present hour the countless societies and colleges of our own and other lands devoted to the healing art, are proof of this. So that wherever social freedom has existed, or tyranny would permit, internal organization and development has been the rule of our profession.

"With these facts before us, our origin and growth as an element of civilization, is a subject worthy of some attention. I propose to occupy the passing hour in contemplating it, so far at least as relates to the condition of medicine in ancient times, and among those people from whom the usages of modern society have been derived.

"This subject is one which has often furnished occupation for my leisure hours. It is pleasant as well as profitable to turn, on fit occasions, from the bustle of active life to the study of the past: to the origin of our art; to the principles and necessities that called it into being; to the struggles of our ancestry. We are thereby better able to understand our own position, to know how far we have advanced, to whom we owe our progress, the labor still before us, and the places we ourselves are likely to occupy in the estimation of those who are to follow us."

A work so purely historical as the present one, scarcely admits of analysis, and must be read to be appreciated. Dr. Watson's style is chaste and direct; and, in choosing such a theme and bestowing upon it so much time and labor, he has set an example we would be glad to see followed everywhere by those appointed to discharge the literary duties imposed by the various medical organizations of our country. The discourse of Dr. Watson is truly an honor to its author, to the Academy of Medicine, and to the literature of our profession.

